# Harmonic Noise-Tolerant ZNN for Dynamic Matrix Pseudoinversion and Its Application to Robot Manipulator

**DOI:** 10.3389/fnbot.2022.928636

**Published:** 2022-06-13

**Authors:** Bolin Liao, Yuyan Wang, Jianfeng Li, Dongsheng Guo, Yongjun He

**Affiliations:** ^1^College of Information Science and Engineering, Jishou University, Jishou, China; ^2^College of Mathematics and Statistics, Jishou University, Jishou, China; ^3^School of Information Science and Engineering, Huaqiao University, Xiamen, China

**Keywords:** zeroing neural network, harmonic noise, matrix pseudoinverse, robot manipulator, robustness

## Abstract

As we know, harmonic noises widely exist in industrial fields and have a crucial impact on the computational accuracy of the zeroing neural network (ZNN) model. For tackling this issue, by combining the dynamics of harmonic signals, two harmonic noise-tolerant ZNN (HNTZNN) models are designed for the dynamic matrix pseudoinversion. In the design of HNTZNN models, an adaptive compensation term is adopted to eliminate the influence of harmonic noises, and a Li activation function is introduced to further improve the convergence rate. The convergence and robustness to harmonic noises of the proposed HNTZNN models are proved through theoretical analyses. Besides, compared with the ZNN model without adaptive compensation term, the HNTZNN models are more effective for tacking the problem of dynamic matrix pseudoinverse under harmonic noises environments. Moreover, HNTZNN models are further applied to the kinematic control of a four-link planar robot manipulator under harmonic noises. In general, the experimental results verify the effectiveness, superiority, and broad application prospect of the models.

## 1. Introduction

The matrix pseudoinverse (i.e., Moore Penrose generalized inverse) is the generalized inverse of a singular matrix or non-square matrix. Similar to the inverse of the matrix, the matrix pseudoinverse is an important subject in the fields of science and engineering. It is considered a powerful formula and design technology for image reduction (Juang and Wu, 2010), signal processing (Van der Veen et al., 1997), linear classifier (Skurichina and Duin, 2002), and associative memory (Zhang et al., 2004). Due to its importance, numerous efforts have been made in the calculation of matrix pseudoinverse in the past few decades. For instance, Perković and Stanimirović (2011) developed an iterative algorithm to estimate the Moore Penrose generalized inverse. Zhou et al. (2002) presented a sequential recursive formula to calculate the pseudo inverse of the matrix based on the famous Greville formula. Courrieu (2008) proposed an algorithm based on full rank Cholesky decomposition for fast calculation of Moore Penrose inverse matrix. However, most of these serial processing algorithms need to be executed in a single sampling period, and when the system order becomes large, these algorithms may fail. Therefore, these studies are neither feasible nor valid for real-time applications (Zhang and Yi, 2011). While the real-time computing of dynamic matrix pseudoinverse problems often exists in industrial applications. For example, the inverse kinematics control problem of a redundant robot manipulator needs to calculate the pseudoinverse of the dynamic matrix in real time (Liao et al., 2016).

Due to the nature of high-speed parallel, distributed processing and the convenience of hardware implementation, neural networks have been used to replace the numerical algorithm of serial processing, and widely used in the fields of scientific and engineering (Jin et al., 2017; Xu et al., 2019; Jafari, 2020; Liu et al., 2020). Scholars have deeply studied many recurrent neural networks and parallel computing schemes for computing scientific problems including matrix pseudoinverse in real time (Li et al., 2021; Kornilova et al., 2022). In particular, a special kind of recurrent neural network termed zeroing neural network (ZNN), has achieved remarkable success in solving dynamic problems (Zhang et al., 2002; Ding et al., 2017; Xiao et al., 2017; Jin, 2021). Ding et al. (2017) investigated a new neural network based on ZNN to obtain the online solution of complex-valued systems of linear equation in a complex domain with higher precision and higher convergence rate. Xiao et al. (2017) presented a ZNN to solve effectively the unified QP problem and applied it to the coordinated path tracking of dual robot manipulators. Specifically, ZNN successfully solved the lag error generated by traditional recurrent neural networks which uses the indefinite error monitoring function. In addition, the traditional ZNN does not have obvious advantages in real-time calculation of large-scale problems due to its slow convergence speed. Thus, based on the essential nonlinear method of ZNN design, Liao and Zhang (2014b) proposed two finite time convergent ZNN models to solve the real time dynamic matrix pseudoinverse effectively.

In industrial applications, harmonic noises exist widely (Du et al., 2017; Karsli and Dondurur, 2018). In addition, any type of signal can be expressed as the superposition of a sinusoidal signal based on the Fourier transform. However, most of the current studies are aimed at constant noises or linear noises, and there is a lack of an adaptive mechanism to suppress harmonic noises. Furthermore, high frequency and large amplitude harmonic noises will seriously affect the calculation accuracy of the ZNN model. Therefore, by incorporating the dynamics of harmonic signals, we design and use the adaptive compensation term to learn harmonic noises and compensate for the influence of harmonic noises adaptively. Moreover, a well-defined activation function is helpful to accelerate the convergence rate of the ZNN model (Xiao et al., 2018). Therefore, on the basis of harmonic noise adaptation, we employ the Li activation function (Li et al., 2013) to speed up the convergence rate of ZNN and finally design two harmonic noise-tolerant ZNN (HNTZNN) models for solving the dynamic matrix pseudoinverse problem in the harmonic-noise environment. Furthermore, these HNTZNN models are applied to the kinematic control of a four-link planar robot manipulator.

The remainder of this article is organized into six sections. Section 2 introduces the problem formulation and preliminaries. HNTZNN models are developed and studied in Section 3. In Section 4, the convergence and noise suppression ability of HNTZNN are proved theoretically. In Section 5, numerical examples are used to prove the efficacy and superiority of HNTZNN models for calculating the dynamic matrix pseudoinverse. Section 6 shows the application of two ZNN models on a four-link planar robot manipulator. Finally, the conclusion of this article is given in Section 7. The main contributions of this article are as follows.

- In this article, two novel harmonic noise-tolerant ZNN models with a fast convergence rate are first proposed and investigated for computing dynamic matrix pseudoinversion by combining the dynamic properties of harmonic signals and the Li activation function.
- Theoretical analyses are conducted, which deduce the excellent convergence and robustness of these HNTZNN models in coping with single-harmonic and multiple-harmonic noises.
- The experiment results are illustrated, which further substantiate the efficacy and superiority of the proposed HNTZNN models for the dynamic matrix pseudoinversion under low-frequency, high-frequency, periodic, and aperiodic harmonic noises.
- The proposed HNTZNN models are applied to the kinematic control of a four-link planar robot manipulator in the presence of harmonic noise, thereby depicting the application prospect of the proposed models.

## 2. Problem Formulation and Preliminaries

**Definition 1**. *(Wang, 1997; Liao and Zhang, 2014a) For a given dynamic matrix* *B*(*t*) ∈ ℝ^*m*×*n*^, *its pseudoinverse* *Y*(*t*) ∈ ℝ^*n*×*m*^ *satisfies the following Penrose equations*:


$$\begin{array}{l} B\left(t\right)Y\left(t\right)B\left(t\right)=B\left(t\right),\;Y\left(t\right)B\left(t\right)Y\left(t\right)=Y\left(t\right),\\ B\left(t\right)Y\left(t\right)={\left(B\left(t\right)Y\left(t\right)\right)}^{\text{T}},\;Y\left(t\right)B\left(t\right)={\left(Y\left(t\right)B\left(t\right)\right)}^{\text{T}}, \end{array}$$

where (·)^T^ denotes the transpose of a matrix, *Y*(*t*) is called the dynamic pseudoinverse of *B*(*t*), which is often denoted by *B*^†^(*t*).

It is worth noting that the dynamic pseudoinverse *B*^†^(*t*) always exists and is unique (Liao and Zhang, 2014a). In particular, if the matrix *B*(*t*) is a full rank matrix at any time instant *t*, i.e., rank[*B*(*t*)] = min{*m, n*} ∀*t* ∈ [0, ∞), the dynamic pseudoinverse of *B*(*t*) is obtained by the following lemma.

**Lemma 1**. *(Wang, 1997; Liao and Zhang, 2014a) For any dynamic matrix* *B*(*t*) ∈ ℝ^*m*×*n*^, *if rank*[*B*(*t*)] = min{*m, n*} ∀*t* ∈ [0, ∞), *the unique dynamic pseudoinverse* *B*^†^(*t*) *can be expressed as*:


$$\begin{array}{l} B^{†}\left(t\right)=\{\begin{array}{cc} {\left(B^{\text{T}}\left(t\right)B\left(t\right)\right)}^{-1}B^{\text{T}}\left(t\right), & \text{if }m>n,\\ B^{\text{T}}\left(t\right){\left(B\left(t\right)B^{\text{T}}\left(t\right)\right)}^{-1}, & \text{if }m\leq n. \end{array} \end{array}$$

When *m* > *n*, it represents the left pseudoinverse of the matrix *B*(*t*). When *m* ≤ *n*, it represents the right pseudoinverse of the matrix *B*(*t*). In the case of *m* ≤ *n*, the procedure of obtaining the dynamic pseudoinverse *B*^†^(*t*) is similar to that of *m* > *n*, and thus this paper only considers the case of the left pseudoinverse of the matrix *B*(*t*). Therefore, the dynamic pseudoinverse problem studied in this study is in the form of


(1) $$\begin{array}{l} B^{\text{T}}\left(t\right)B\left(t\right)Y\left(t\right)=B^{\text{T}}\left(t\right)\in {\mathbb{R}}^{m\times n}. \end{array}$$

Our objective in this study is to find the *Y*(*t*) of the problem (1).

## 3. Harmonic Noise-Tolerant ZNN Models

In this section, the improved ZNN models against harmonic noises for dynamic pseudoinverse are detailed.

### 3.1. ZNN Model Against Single-Harmonic Noise

In order to monitor the calculation process of dynamic matrix pseudoinverse (1) in real time, the error function is defined as


$$\begin{array}{l} E\left(t\right)=B^{\text{T}}\left(t\right)B\left(t\right)Y\left(t\right)-B^{\text{T}}\left(t\right). \end{array}$$

To force *E*(*t*) to converge to zero, we choose


(2) $$\begin{array}{l} Ė\left(t\right)=\frac{\text{d}E\left(t\right)}{\text{d}t}=-\tau \text{Ψ}(E\left(t\right)), \end{array}$$

where τ > 0 ∈ ℝ is a positive design parameter, and Ψ(·) denotes the activation function of the neural network, which is a monotonically increasing odd function. Then,


(3) $$\begin{array}{l} Ė\left(t\right)=B^{\text{T}}\left(t\right)B\left(t\right)Ẏ\left(t\right)\\ \;+({Ḃ}^{\text{T}}\left(t\right)B\left(t\right)+B^{\text{T}}\left(t\right)Ḃ\left(t\right))Y\left(t\right)-{Ḃ}^{\text{T}}\left(t\right). \end{array}$$

In this article, we choose the Li activation function (Li et al., 2013), and its expression is as follows:


(4) $$\begin{array}{l} \psi \left(e_{ij}\right)={\text{Lip}}^{\lambda }\left(e_{ij}\right)+{\text{Lip}}^{1/\lambda }\left(e_{ij}\right), \end{array}$$

where *e*_*ij*_ represents the *ij*th element of *E*(*t*), parameter λ ∈ (0, 1), and the function Lip^λ^(·) is defined as follows:


(5) $$\begin{array}{l} {\text{Lip}}^{\lambda }\left(e_{ij}\right)=\{\begin{array}{cc} |e_{ij}{|}^{\lambda }, & \text{if }e_{ij}>0,\\ 0, & \text{if }e_{ij}=0,\\ -|e_{ij}{|}^{\lambda }, & \text{if }e_{ij}<0. \end{array} \end{array}$$

In view of the noise exiting, formula (2) can be rewritten as


(6) $$\begin{array}{l} Ė\left(t\right)=-\tau \text{Ψ}(E\left(t\right))+O\left(t\right), \end{array}$$

where *O*(*t*) ∈ ℝ^*m*×*n*^ represents the matrix-form harmonic noises and each element is defined as follows:


(7) $$\begin{array}{l} o_{ij}\left(t\right)=\Gamma \sin\left(\omega t+\varphi \right)=\Gamma \sin\left(2\pi ft+\varphi \right), \end{array}$$

where *i* ∈ {1, ⋯ , *m*}, *j* ∈ {1, ⋯ , *n*}. It is assumed that the frequency *f* in (7) is known, the amplitude Γ and phase φ are unknown. To adaptively learn the *f*-frequency harmonics noise, an additional quantity −*X*(*t*) ∈ ℝ^*m*×*n*^ is introduced into equation (6):


(8) $$\begin{array}{l} Ė\left(t\right)=-\tau \text{Ψ}(E\left(t\right))-X\left(t\right)+O\left(t\right). \end{array}$$

The purpose of this study is to design and use −*X*(*t*) to adaptively learn *O*(*t*) and compensate for the impact of *O*(*t*). Differentiating (7) twice with respect to time *t*, the following result is obtained:


$$\begin{array}{l} {ö}_{ij}\left(t\right)=-4\pi^{2}f^{2}\Gamma \sin\left(2\pi ft+\varphi \right)=-4\pi^{2}f^{2}o_{ij}\left(t\right). \end{array}$$

Thus, the dynamics of *o*_*ij*_(*t*) can be formulated as follows:


(9) $$\begin{array}{l} {ȯ}_{ij}\left(t\right)=n_{ij}\left(t\right),{ṅ}_{ij}\left(t\right)=-4\pi^{2}f^{2}o_{ij}\left(t\right). \end{array}$$

Rewrite formula (9) into matrix form:


(10) $$\begin{array}{l} Ȯ\left(t\right)=N\left(t\right),Ṅ\left(t\right)=-4\pi^{2}f^{2}O\left(t\right), \end{array}$$

where *N*(*t*) ∈ ℝ^*m*×*n*^ with *n*_*ij*_(*t*) as its element. The unknown amplitude and phase information of a harmonic signal can be adaptively eliminated from (10) when the frequency *f* is known.

Therefore, combing formulas (3), (8), and (10), the following single-harmonic noise-tolerant ZNN (HNTZNN) model is obtained:


(11) $$\begin{array}{l} \{\begin{array}{c} B^{\text{T}}\left(t\right)B\left(t\right)Ẏ\left(t\right)={Ḃ}^{\text{T}}\left(t\right)-({Ḃ}^{\text{T}}\left(t\right)B\left(t\right)+B^{\text{T}}\left(t\right)Ḃ\left(t\right))Y\left(t\right)\\ -\tau \text{Ψ}(B^{\text{T}}\left(t\right)B\left(t\right)Y\left(t\right)-B^{\text{T}}\left(t\right))-X\left(t\right)+O\left(t\right),\\ Ẋ\left(t\right)=C\left(t\right)+4\pi^{2}f^{2}\lambda E\left(t\right),\\ Ċ\left(t\right)=-4\pi^{2}f^{2}X\left(t\right), \end{array} \end{array}$$

where λ ≥ 1 ∈ ℝ.

### 3.2. ZNN Model Against Multiple-Harmonic Noise

In this section, the proposed HNTZNN model (11) is extended to multiple-harmonic noises, and a neural network model against multiple-harmonic noises is obtained. Let Õ(*t*) ∈ ℝ^*m*×*n*^ be the matrix-form multiple-harmonic noises. Each element of such matrix is defined as


(12) $$\begin{array}{l} {õ}_{ij}\left(t\right)=\overset{l}{\underset{k=1}{\sum }}o_{k}\left(t\right)=\overset{l}{\underset{k=1}{\sum }}\Gamma_{k}\sin\left(2\pi f_{k}t+\varphi_{k}\right). \end{array}$$

Similarly, in (12), it is assumed that the frequency *f*_*k*_ (with *k* ∈ 1, ⋯ , *l*) is known, the amplitude Γ_*k*_ and phase φ_*k*_ are unknown.

Differentiating (12) twice with respect to time *t*, we have the following results:


$$\begin{array}{l} \dot{õ}\left(t\right)=\overset{l}{\underset{k=1}{\sum }}{ö}_{k}\left(t\right)\\ \;=\overset{l}{\underset{k=1}{\sum }}-4\pi^{2}f_{k}^{2}\Gamma_{k}\sin\left(2\pi f_{k}t+\varphi_{k}\right)\\ \;=\overset{l}{\underset{k=1}{\sum }}-4\pi^{2}f_{k}^{2}o_{k}\left(t\right). \end{array}$$

The dynamics of õ_*i*_*j*(*t*) are obtained as follows:


$$\begin{array}{l} {\dot{õ}}_{ij}\left(t\right)=\overset{l}{\underset{k=1}{\sum }}n_{k}\left(t\right),{ȯ}_{k}\left(t\right)=n_{k}\left(t\right),{ṅ}_{k}\left(t\right)=-4\pi^{2}f_{k}^{2}o_{k}\left(t\right), \end{array}$$

which is rewritten in the matrix form as:


(13) $$\begin{array}{l} \dot{Õ}\left(t\right)=\overset{l}{\underset{k=1}{\sum }}N_{k}\left(t\right),{Ȯ}_{k}\left(t\right)=N_{k}\left(t\right),{Ṅ}_{k}\left(t\right)=-4\pi^{2}{f_{k}}^{2}N_{k}\left(t\right), \end{array}$$

where $N_{k}\left(t\right)\in {\mathbb{R}}^{m\times n}$ and $O_{k}\left(t\right)\in {\mathbb{R}}^{m\times n}$ are the matrices composed of *n*_*k*_(*t*) and *o*_*k*_(*t*), respectively.

Combined with the dynamic analysis in Formula (13), a multiple-harmonic noise-tolerant ZNN model is as follows:


(14) $$\begin{array}{l} \{\begin{array}{c} B^{\text{T}}\left(t\right)B\left(t\right)Ẏ\left(t\right)={Ḃ}^{\text{T}}\left(t\right)-({Ḃ}^{\text{T}}\left(t\right)B\left(t\right)+B^{\text{T}}\left(t\right)Ḃ\left(t\right))Y\left(t\right)\\ -\tau \text{Ψ}(B^{\text{T}}\left(t\right)B\left(t\right)Y\left(t\right)-B^{\text{T}}\left(t\right))-\sum_{k=1}^{l}X_{k}\left(t\right)+Õ\left(t\right),\\ \dot{X_{k}}\left(t\right)=C_{k}\left(t\right)+4\pi^{2}f_{k}^{2}\lambda E\left(t\right),\\ \dot{C_{k}}\left(t\right)=-4\pi^{2}f_{k}^{2}X_{k}\left(t\right),k=1,2,\cdots \;,l. \end{array} \end{array}$$

When *l* = 1, (14) can be reduced to (11).

The corresponding theoretical analysis and proof are carried out in the next section.

## 4. Theoretical Analysis

Three theorems are proposed to verify the convergence and robustness performance of the proposed HNTZNN model.

**Theorem 1**. *For any smooth dynamic full rank matrix* *B*(*t*) ∈ ℝ^*m*×*n*^ (*m* > *n*), *the state matrix* *Y*(*t*) *of HNTZNN model (11) globally converges to the dynamic theoretical pseudoinverse* *B*^†^(*t*) *of (1) in the free-noise situation*.

Proof: In the free-noise situation, (11) can be simplified as


(15) $$\begin{array}{l} \{\begin{array}{c} cĖ\left(t\right)=-\tau \text{Ψ}(E\left(t\right))-X\left(t\right),\\ Ẋ\left(t\right)=C\left(t\right)+4\pi^{2}f^{2}\lambda E\left(t\right),\\ Ċ\left(t\right)=-4\pi^{2}f^{2}X\left(t\right), \end{array} \end{array}$$

where *E*(*t*) = *B*^T^(*t*)*B*(*t*)*Y*(*t*) − *B*^T^(*t*), (15) is a compact matrix form of the following set of *mn*-decoupled equations:


(16) $$\begin{array}{l} \{\begin{array}{c} c{ė}_{ij}\left(t\right)=-\tau \psi (e_{ij}\left(t\right))-x_{ij}\left(t\right),\\ {ẋ}_{ij}\left(t\right)=c_{ij}\left(t\right)+4\pi^{2}f^{2}\lambda e_{ij}\left(t\right),\\ {ċ}_{ij}\left(t\right)=-4\pi^{2}f^{2}x_{ij}\left(t\right). \end{array} \end{array}$$

Then, we could define the Lyapunov function candidate (Xiang et al., 2019) to analyze the *ij*th subsystem (16) as


(17) $$\begin{array}{l} v_{ij}\left(t\right)=\frac{e_{ij}^{2}\left(t\right)}{2}+\frac{x_{ij}^{2}\left(t\right)}{8\pi^{2}f^{2}\lambda }+\frac{c_{ij}^{2}\left(t\right)}{32\pi^{4}f^{4}\lambda }, \end{array}$$

which guarantees *v*_*ij*_(*t*) ≥ 0, i.e., *v*_*ij*_(*t*) > 0 for any $e_{ij}^{2}\left(t\right)\neq 0$ or $x_{ij}^{2}\left(t\right)\neq 0$ or $c_{ij}^{2}\left(t\right)\neq 0$, and *v*_*ij*_(*t*) = 0 if and only if $e_{ij}^{2}\left(t\right)=0$, $x_{ij}^{2}\left(t\right)=0$, $c_{ij}^{2}\left(t\right)=0$. The time derivative of (17) could be obtained as


$$\begin{array}{l} \frac{\text{d}v_{ij}\left(t\right)}{\text{d}t}=e_{ij}\left(t\right){ė}_{ij}\left(t\right)+\frac{x_{ij}\left(t\right){ẋ}_{ij}\left(t\right)}{4\pi^{2}f^{2}\lambda }+\frac{c_{ij}\left(t\right){ċ}_{ij}\left(t\right)}{16\pi^{4}f^{4}\lambda },\\ \;=-\tau e_{ij}\left(t\right)\psi (e_{ij}\left(t\right))-e_{ij}\left(t\right)x_{ij}\left(t\right)+\frac{x_{ij}\left(t\right)c_{ij}\left(t\right)}{4\pi^{2}f^{2}\lambda }\\ \;+x_{ij}\left(t\right)e_{ij}\left(t\right)-\frac{c_{ij}\left(t\right)x_{ij}\left(t\right)}{4\pi^{2}f^{2}\lambda },\\ \;=-\tau e_{ij}\left(t\right)\psi (e_{ij}\left(t\right)). \end{array}$$

Substituting (5) into the above equation, we further have


$$\begin{array}{l} \frac{\text{d}v_{ij}\left(t\right)}{\text{d}t}=\{\begin{array}{cc} -\tau |e_{ij}\left(t\right){|}^{\lambda +1},\; & \text{if }e_{ij}\neq 0,\\ 0,\; & \text{if }e_{ij}=0. \end{array} \end{array}$$

Thus, when *e*_*ij*_(*t*) ≠ 0, ${\dot{v}}_{ij}\left(t\right)<0$, when *e*_*ij*_(*t*) = 0, ${\dot{v}}_{ij}\left(t\right)=0$. According to Lyapunov stability theory (Khalil, 2001), it can be concluded that *e*_*ij*_(*t*) globally to 0 for any *i* ∈ {1, ⋯ , *m*}, *j* ∈ {1, ⋯ , *n*}, which means that


$$\begin{array}{l} \underset{t\to \infty }{\lim}{\left\|E\left(t\right)\right\|}_{\text{F}}=0. \end{array}$$

In view of *E*(*t*) = *B*^T^(*t*)*B*(*t*)*Y*(*t*) − *B*^T^(*t*) and the nonsingularity of *B*^T^(*t*)*B*(*t*), we can deduce *Y*(*t*) → (*B*^T^(*t*)*B*(*t*))^−1^*B*^T^(*t*) as *t* → ∞. Therefore, the state matrix *Y*(*t*) of HNTZNN model (11) globally converges to the dynamic theoretical pseudoinverse *B*^†^(*t*) of (1). The proof is, thus, completed.

**Theorem 2**. *For any smooth dynamic full rank matrix* *B*(*t*) ∈ ℝ^*m*×*n*^ (*m* > *n*), *the state matrix* *Y*(*t*) *of HNTZNN model (11) globally converges to the dynamic theoretical pseudoinverse* *B*^†^(*t*) *of (1) when the single-harmonic noise is considered*.

Proof: Let us consider a single harmonic noise *O*(*t*) = [*o*_*ij*_(*t*)] = [Γsin(2π*ft* + φ)] with known frequency, and the unknown amplitude and phase. Model (11) is simplified and rewritten as


(18) $$\begin{array}{l} \{\begin{array}{c} cĖ\left(t\right)=-\tau \text{Ψ}(E\left(t\right))-X\left(t\right)+O\left(t\right),\\ Ẋ\left(t\right)=C\left(t\right)+4\pi^{2}f^{2}\lambda E\left(t\right),\\ Ċ\left(t\right)=-4\pi^{2}f^{2}X\left(t\right),\\ Ȯ\left(t\right)=N\left(t\right),\\ Ṅ\left(t\right)=-4\pi^{2}f^{2}O\left(t\right). \end{array} \end{array}$$

Defining Δ(*t*) = *X*(*t*) − *O*(*t*) and Φ(*t*) = *C*(*t*) − *N*(*t*) yields the following reformulation of (18):


(19) $$\begin{array}{l} \{\begin{array}{c} Ė\left(t\right)=-\tau \text{Ψ}(E\left(t\right))-\Delta \left(t\right),\\ \dot{\Delta }\left(t\right)=\Phi \left(t\right)+4\pi^{2}f^{2}\lambda E\left(t\right),\\ \dot{\Phi }\left(t\right)=-4\pi^{2}f^{2}\Delta \left(t\right). \end{array} \end{array}$$

Noting that Equations (15) and (19) have exactly the same form, therefore, one can also obtain


$$\begin{array}{l} \underset{t\to \infty }{\lim}e_{ij}\left(t\right)=0\;\text{and}\underset{t\to \infty }{\lim}{\left\|E\left(t\right)\right\|}_{\text{F}}=0, \end{array}$$

for any *i* ∈ {1, ⋯ , *m*}, *j* ∈ {1, ⋯ , *n*}. In addition, according to (16), we have


(20) $$\begin{array}{l} \{\begin{array}{c} c{ẋ}_{ij}\left(t\right)=c_{ij}\left(t\right)+4\pi^{2}f^{2}\lambda e_{ij}\left(t\right)\to c_{ij}\left(t\right),\\ {ċ}_{ij}\left(t\right)=-4\pi^{2}f^{2}x_{ij}\left(t\right). \end{array} \end{array}$$

According to the dynamic description in (9), Equation (20) can generate a harmonic signal *x*_*ij*_(*t*) that can adaptively compensate for the influence of noise over time. Then, Equation (18) is a compact matrix form of the following set of *mn*-decoupled equations:


(21) $$\begin{array}{l} \{\begin{array}{c} c{ė}_{ij}\left(t\right)=-\tau \psi (e_{ij}\left(t\right))-x_{ij}\left(t\right)+o_{ij}\left(t\right),\\ {ẋ}_{ij}\left(t\right)=c_{ij}\left(t\right)+4\pi^{2}f^{2}\lambda e_{ij}\left(t\right),\\ {ċ}_{ij}\left(t\right)=-4\pi^{2}f^{2}x_{ij}\left(t\right). \end{array} \end{array}$$

Because of $\lim_{t\to \infty }e_{ij}\left(t\right)=0$, the harmonic signal *x*_*ij*_(*t*) is generated based on the analysis of (20), which meets the following results:


$$\begin{array}{l} -x_{ij}\left(t\right)+o_{ij}\left(t\right)\to 0,\;\text{as }t\to \infty . \end{array}$$

In particular, although only the frequency of *o*_*ij*_(*t*) is known, the second and third dynamics in (21) can adaptively predict the unknown parameters of *o*_*ij*_(*t*), i.e., amplitude Γ and phase φ. The resultant signal *x*_*ij*_(*t*) can adaptively compensate the impact of *o*_*ij*_(*t*).

In summary, the state matrix *Y*(*t*) of the HNTZNN model (11) globally converges to the dynamic theoretical pseudoinverse *B*^†^(*t*) of (1) when the single-harmonic noise is considered. The proof is, thus, completed.

**Theorem 3**. *For any smooth dynamic full rank matrix* *B*(*t*) ∈ ℝ^*m*×*n*^ (*m* > *n*), *the state matrix* *Y*(*t*) *of the HNTZNN model (14) globally converges to the dynamic theoretical pseudoinverse* *B*^†^(*t*) *of (1) when the multiple-harmonic noise is considered, i.e.,* $Õ\left(t\right)=\left[o_{ij}\left(t\right)\right]=\left[\sum_{k=1}^{l}\Gamma_{k}\sin\left(2\pi f_{k}t+\varphi_{k}\right)\right]$, *i* ∈ {1, ⋯ , *m*}, *j* ∈ {1, ⋯ , *n*}.

Proof: This theorem can be obtained through the proof of the above two theorems and the superposition principle. Therefore, it is omitted. The proof is, thus, completed.

*Remarks:* Generally, for any periodic noises which can be decomposed into a series of harmonics through Fourier transform, the proposed model is effective. The limitation of the current work may lie in the suppression of random noises.

## 5. Numerical Verifications

Previously, we analyzed the convergence and noise tolerance of models (11) and (14). In this section, we verify the efficacy and the superiority of the proposed models for solving the following dynamic matrix pseudoinverse:


(22) $$\begin{array}{l} B\left(t\right)=\left[\begin{array}{cc} \sin\left(2t\right) & \cos\left(2t\right)\\ -\cos\left(2t\right) & \sin\left(2t\right)\\ \sin\left(2t\right) & \cos\left(2t\right) \end{array}\right]\in {\mathbb{R}}^{3\times 2}. \end{array}$$

To check the correctness of the presented HNTZNN models, the theoretical dynamic matrix pseudoinverse of (22) is directly given as


(23) $$\begin{array}{l} B^{†}\left(t\right)=\left[\begin{array}{ccc} 0.5\sin\left(2t\right) & -\cos\left(2t\right) & 0.5\sin\left(2t\right)\\ 0.5\cos\left(2t\right) & \sin\left(2t\right) & 0.5\cos\left(2t\right) \end{array}\right]\in {\mathbb{R}}^{2\times 3}. \end{array}$$

In order to verify the superiority of the proposed model, the ZNN-1 model (Liao and Zhang, 2014a) for dynamic matrix pseudoinverse is directly given in this section:


(24) $$\begin{array}{l} B^{\text{T}}\left(t\right)B\left(t\right)Ẏ\left(t\right)={Ḃ}^{\text{T}}\left(t\right)-({Ḃ}^{\text{T}}\left(t\right)B\left(t\right)+B^{\text{T}}\left(t\right)Ḃ\left(t\right))Y\left(t\right)\\ \;-\tau \text{Ψ}(B^{\text{T}}\left(t\right)B\left(t\right)Y\left(t\right)-B^{\text{T}}\left(t\right)). \end{array}$$

The following experiments are carried out to solve the pseudoinverse of the dynamic matrix (22) in the case of four kinds of harmonic noise.

**Figure 1:**
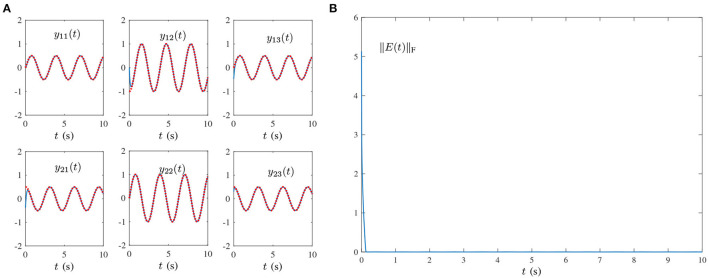
Results synthesized by the HNTZNN model (11) using τ = 10 and λ = 1 for computing the pseudoinverse of the dynamic matrix (21) under the single-harmonic noise with frequency *f* = 0.001 Hz. **(A)** State trajectory. **(B)** Residual error.

**Figure 2:**
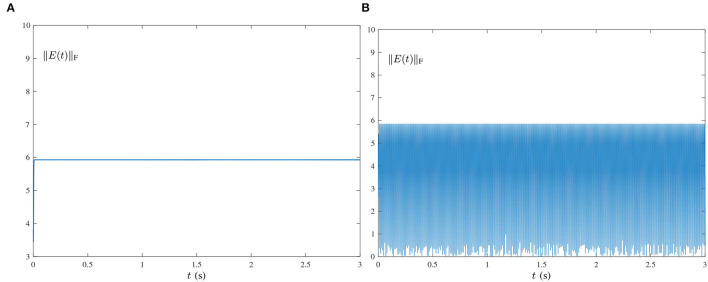
Residual errors of ZNN-1 model (24) for computing the pseudoinverse of the dynamic matrix (21) under the single-harmonic noise with different frequencies. **(A)** *f* = 0.001 Hz. **(B)** *f* = 50 Hz.

**Figure 3:**
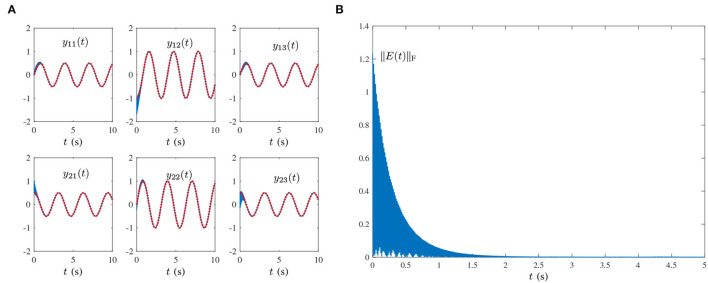
Results synthesized by the HNTZNN model (11) using τ = 10 and λ = 1 for computing the pseudoinverse of the dynamic matrix (21) under the single-harmonic noise with frequency *f* = 50 Hz. **(A)** State trajectory. **(B)** Residual error.

**Figure 4:**
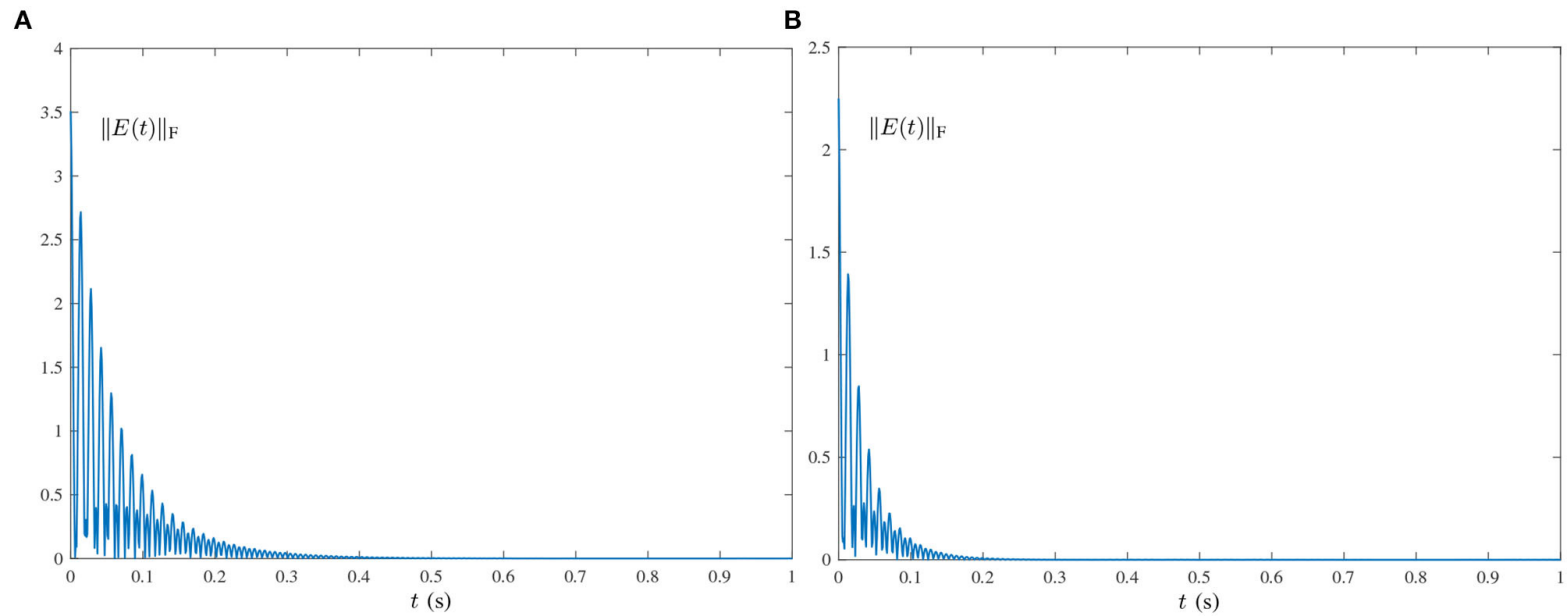
Residual errors of HNTZNN model (11) using different τ for computing the pseudoinverse of the dynamic matrix (21) under the single-harmonic noise with frequency *f* = 50 Hz. **(A)** τ = 50, λ = 1. **(B)** τ = 100, λ = 1.

**Figure 5:**
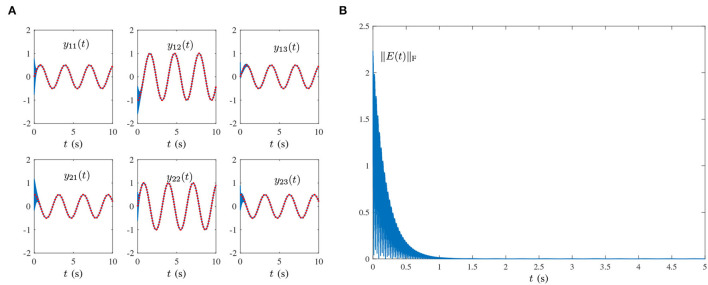
Results synthesized by the HNTZNN model (14) using τ = 10 and λ = 1 for computing the pseudoinverse of the dynamic matrix (21) under the multiple-harmonic noise with a periodic characteristic. **(A)** State trajectory. **(B)** Residual error.

**Figure 6:**
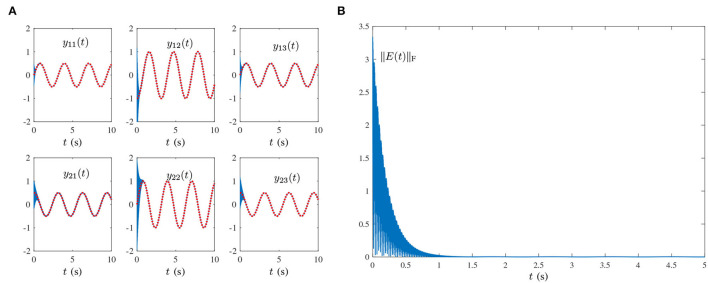
Results synthesized by the HNTZNN model (14) using τ = 10 and λ = 1 for computing the pseudoinverse of the dynamic matrix (21) under the multiple-harmonic noise with an aperiodic characteristic. **(A)** State trajectory. **(B)** Residual error.

1. Single-harmonic noise with a low-frequency: Figures 1, 2 present the experimental result synthesized by HNTZNN model (11) and ZNN-1 model (24) for the dynamic matrix (21) pseudoinverse under the single-harmonic noise with a near-zero frequency, i.e.,
  $$\begin{array}{l} O\left(t\right)=\left[o_{ij}\left(t\right)\right]=\left[10^{3}\sin\left(0.002\pi t+1\right)\right]\in {\mathbb{R}}^{2\times 3}, \end{array}$$
  where frequency *f* = 0.002π/(2π) = 0.001Hz. In Figure 1A, the red dashed-dotted line denotes the theoretical dynamic solution, and the blue solid line denotes the neural-state solution. In addition, we set τ = 10 and λ = 1. As shown in Figure 1A, for a random initial value *Y*(0) ∈ ℝ^2×3^, state matrix *Y*(*t*) ∈ ℝ^2×3^ of the proposed HNTZNN model (11) converges to the theoretical pseudoinverse (23) accurately and rapidly in a short period of time. Figure 1B shows that the residual error ‖*E*(*t*)‖_F_ of the HNTZNN model (11) can converge to zero within 1 s. However, with the same parameters, the residual error ‖*E*(*t*)‖_F_ of the ZNN-1 model (24) can not converge to zero displayed in Figure 2A. These experimental results verify the efficacy and superiority of model (11) for solving the dynamic matrix pseudoinverse under the single-harmonic noise.
2. Single-harmonic noise with high-frequency: HNTZNN model (11) and ZNN-1 model (24) are tested for the following harmonic noise:
  $$\begin{array}{l} O\left(t\right)=\left[o_{ij}\left(t\right)\right]=\left[10^{3}\sin\left(100\pi t+1\right)\right]\in {\mathbb{R}}^{2\times 3}, \end{array}$$
  where frequency *f* = 50 Hz, which is widely encountered in power systems (Karsli and Dondurur, 2018). The corresponding results are shown in Figures 2, 3. As shown in Figure 3A, the state matrix *Y*(*t*) ∈ ℝ^2×3^ of the proposed HNTZNN model (11) still can converge to the dynamic theoretical pseudoinverse (23) accurately and rapidly. Figure 3B shows that the residual error ‖*E*(*t*)‖_F_ of HNTZNN model (11) can converge to zero rapidly. On the contrary, Figure 2B shows that the residual error ‖*E*(*t*)‖_F_ of ZNN-1 model (24) has a large value under harmonic noise and cannot converge to zero.
  For further investigation, let λ = 1 and vary the value of τ (i.e., let τ = 50 and τ = 100). The experimental results are shown in Figure 4. It can be seen from Figures 3B, 4 that the larger value of τ, the faster convergence rate of HNTZNN model (11).
3. Multiple-harmonic noise with a periodic characteristic: HNTZNN model (14) is tested for the following harmonic noise:
  $$\begin{array}{l} Õ\left(t\right)=\left[{õ}_{ij}\left(t\right)\right]\\ =\left[\sin\left(2\pi t+1\right)+2\sin\left(4\pi t+2\right)+5\sin\left(10\pi t+4\right)\right]\in {\mathbb{R}}^{2\times 3}, \end{array}$$
  where frequencies are denoted by *f*_1_ = 1, *f*_2_ = 2, *f*_3_ = 5 Hz. As shown in Figure 5, the state matrix *Y*(*t*) of proposed HNTZNN model (14) converges to the dynamic theoretical pseudoinverse (23), and the residual error ‖*E*(*t*)‖_F_ of HNTZNN model (11) is convergent to zero.
4. Multiple-harmonic noise with an aperiodic characteristic: HNTZNN model (14) is tested for the following harmonic noise:
  $$\begin{array}{l} Õ\left(t\right)=\left[{õ}_{ij}\left(t\right)\right]\\ \;=\left[\sin\left(1\right)+2\sin\left(5t+2\right)+5\sin\left(10\pi t+3\right)\right]\in {\mathbb{R}}^{2\times 3}, \end{array}$$
  where frequencies are denoted by *f*_1_ = 0Hz, *f*_2_ = 2.5/πHz, *f*_3_ = 5Hz. The corresponding results are demonstrated in Figure 6. As shown in Figure 6, the state matrix *Y*(*t*) of proposed HNTZNN model (14) converges to the dynamic theoretical pseudoinverse (23), and the residual error ‖*E*(*t*)‖_F_ of HNTZNN model (11) is convergent to zero.
  In summary, the experimental results verify the effectiveness and superiority of HNTZNN models (11) and (14) for solving the dynamic matrix pseudoinverse in the presence of harmonic noises.

## 6. Robot Manipulator Application

In this section, HNTZNN models (11) and (14) are applied to the kinematic control of a four-link planar robot manipulator under the presence of harmonic noises.

### 6.1. ZNN-Combined Kinematic Control

The geometry of the four-link planar robot manipulator is shown in Guo et al. (2018a). The Jacobian matrix *J*(θ(*t*)) ∈ ℝ^2×4^ of this robot manipulator is:


$$\begin{array}{l} J\left(\theta \left(t\right)\right)=\left[\begin{array}{cccc} -\overset{4}{\underset{i=1}{\sum }}l_{i}s_{i} & -\overset{4}{\underset{i=2}{\sum }}l_{i}s_{i} & -\overset{4}{\underset{i=3}{\sum }}l_{i}s_{i} & -l_{i}s_{i}\\ -\overset{4}{\underset{i=1}{\sum }}l_{i}c_{i} & -\overset{4}{\underset{i=2}{\sum }}l_{i}c_{i} & -\overset{4}{\underset{i=3}{\sum }}l_{i}c_{i} & -l_{i}c_{i} \end{array}\right], \end{array}$$

where θ(*t*) ∈ ℝ^4^ denotes the joint-angle vector, *l*_*i*_ (with *i* = 1, 2, 3, 4) denotes the length of the *i*th link, $s_{i}=\sin\left(\sum_{j=1}^{i}\theta_{j}\right)$, and $c_{i}=\cos\left(\sum_{j=1}^{i}\theta_{j}\right)$. The redundancy-resolution problem of such a robot manipulator, which corresponds to the kinematic control, is described as follows. Given the desired end-effector path $r_{\text{d}}\left(t\right)\in {\mathbb{R}}^{2}$, we need to obtain θ(*t*) in real time *t*. In particular, the following acceleration-level redundancy-resolution problem is formulated by adopting the solutions presented in previous work (Siciliano et al., 2009; Guo et al., 2018b):


(25) $$\begin{array}{l} J\left(t\right)\ddot{\theta }\left(t\right)={\ddot{r}}_{\text{a}}\left(t\right), \end{array}$$

where $\ddot{\theta }\left(t\right)\in {\mathbb{R}}^{4}$ is the joint-acceleration vector, $r_{\text{a}}\left(t\right)\in {\mathbb{R}}^{2}$ denotes actual end-effector path and ${\ddot{r}}_{\text{a}}\left(t\right)={\ddot{r}}_{\text{d}}\left(t\right)-\dot{J}\left(t\right)\dot{\theta }\left(t\right)+k_{1}\left({ṙ}_{\text{d}}\left(t\right)-J\left(t\right)\dot{\theta }\left(t\right)\right)+k_{2}\left(r_{\text{d}}\left(t\right)-\psi \left(\theta \left(t\right)\right)\right)$ with $\dot{\theta }\left(t\right)\in {\mathbb{R}}^{4}$ as the joint-velocity vector and $\dot{J}\left(t\right)$ as the time derivative of the Jacobian matrix *J*(*t*). In addition, *k*_1_ and *k*_2_ > 0 are the feedback gains, ψ(·) is a differentiable nonlinear mapping (Siciliano et al., 2009; Guo et al., 2018b), and ṙ_d_(*t*) and ${\ddot{r}}_{\text{d}}\left(t\right)$ are first-order and second-order time derivatives of *r*_d_(*t*), respectively. By effectively solving the acceleration-level kinematic equation in (25), the purpose of kinematic control for robot manipulator is achieved.

According to Siciliano et al. (2009), the following pseudoinverse-type (P-type) scheme, which is a typical solution to (25), is presented for the four-link planar robot manipulator:


(26) $$\begin{array}{l} \ddot{\theta }\left(t\right)=J^{†}\left(t\right){\ddot{r}}_{\text{a}}\left(t\right)={\left(J^{\text{T}}J\left(t\right)\right)}^{-1}J^{\text{T}}\left(t\right){\ddot{r}}_{\text{a}}\left(t\right). \end{array}$$

The dynamic matrix inversion is integrated into (26) to achieve the kinematic control of the four-link planar robot manipulator. By defining *B*(*t*) = *J*(*t*), combining (11) with (26) yields the following the new ZNN-combined kinematic control method that is tolerant to single-harmonic noise:


(27) $$\begin{array}{l} \{\begin{array}{c} \ddot{\theta }\left(t\right)=B^{†}\left(t\right){\ddot{r}}_{\text{a}}\left(t\right),\\ B^{\text{T}}\left(t\right)B\left(t\right)Ẏ\left(t\right)={Ḃ}^{\text{T}}\left(t\right)-({Ḃ}^{\text{T}}\left(t\right)B\left(t\right)+B^{\text{T}}\left(t\right)Ḃ\left(t\right))Y\left(t\right)\\ -\tau \text{Ψ}(B^{\text{T}}\left(t\right)B\left(t\right)Y\left(t\right)-B^{\text{T}}\left(t\right))-X\left(t\right)+O\left(t\right),\\ Ẋ\left(t\right)=C\left(t\right)+4\pi^{2}f^{2}\lambda E\left(t\right),\\ Ċ\left(t\right)=-4\pi^{2}f^{2}X\left(t\right). \end{array} \end{array}$$

Furthermore, by combining (14) with (26), the new ZNN-combined kinematic control method for multiple-harmonic noise can be obtained as follows:


(28) $$\begin{array}{l} \{\begin{array}{c} \ddot{\theta }\left(t\right)=B^{†}\left(t\right){\ddot{r}}_{\text{a}}\left(t\right),\\ B^{\text{T}}\left(t\right)B\left(t\right)Ẏ\left(t\right)={Ḃ}^{\text{T}}\left(t\right)-({Ḃ}^{\text{T}}\left(t\right)B\left(t\right)+B^{\text{T}}\left(t\right)Ḃ\left(t\right))Y\left(t\right)\\ -\tau \text{Ψ}(B^{\text{T}}\left(t\right)B\left(t\right)Y\left(t\right)-B^{\text{T}}\left(t\right))-\sum_{k=1}^{l}X_{k}\left(t\right)+Õ\left(t\right),\\ \dot{X_{k}}\left(t\right)=C_{k}\left(t\right)+4\pi^{2}f_{k}^{2}\lambda E\left(t\right),\\ \dot{C_{k}}\left(t\right)=-4\pi^{2}f_{k}^{2}X_{k}\left(t\right),k=1,2,\cdots \;,l. \end{array} \end{array}$$

**Figure 7:**
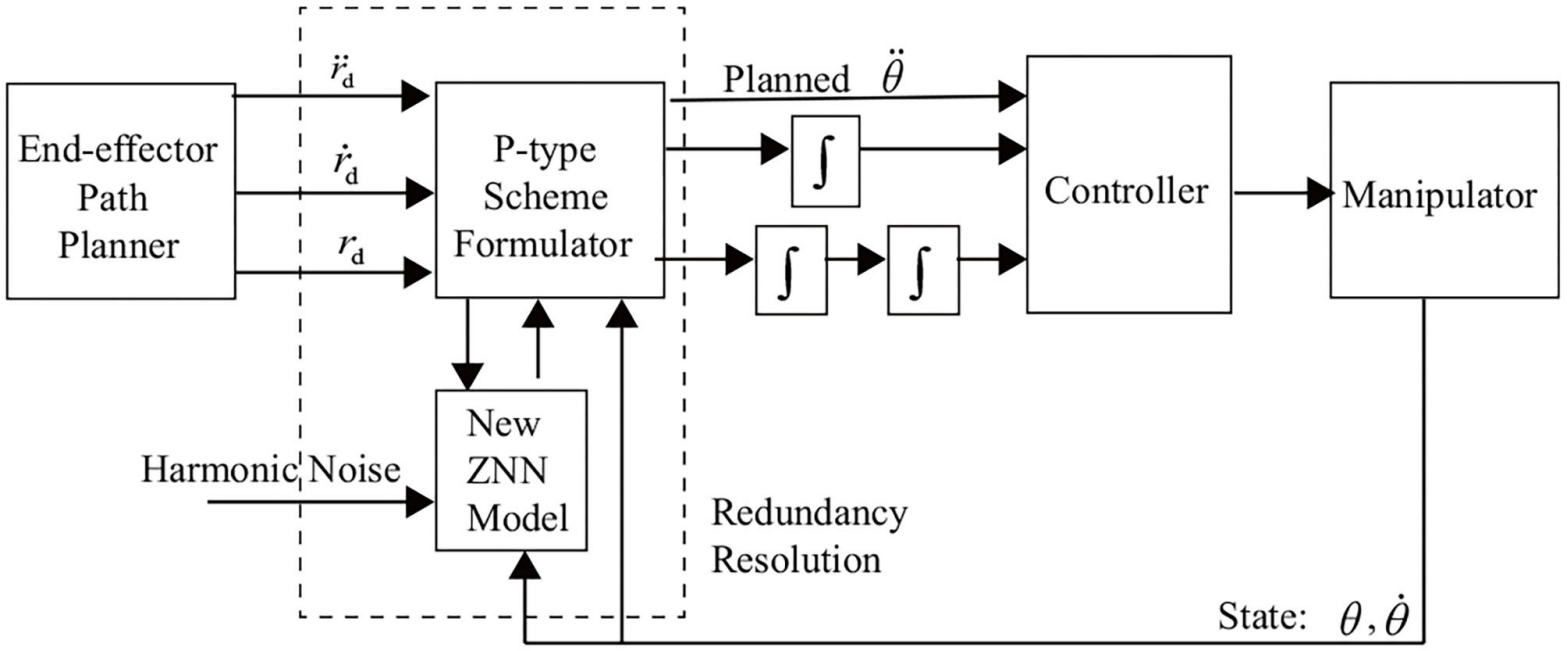
Block diagram of the new ZNN-combined kinematic control method for the four-link planar root manipulator.

To provide a better understanding, we show Figure 7 to depict the block diagram of the ZNN-combined kinematic control method in (28) [including (27) as a special case] for the four-link planar robot manipulator. The robot application in this study is clearly limited by the harmonic noise associated with the ZNN model instead of that of the robot manipulator.

### 6.2. Simulation Verification

In this subsection, the simulation results are presented to show the effectiveness of the presented ZNN-combined kinematic control methods in (27) and (28) for the four-link planar robot manipulator. In the simulations, the lengths of the robot's links are *l*_1_ = *l*_2_ = *l*_3_ = *l*_4_ = 1 m, and the initial joint state is set to θ(0) = [π/9, π/12, π/12, π/12]^T^ rad.

**Figure 8:**
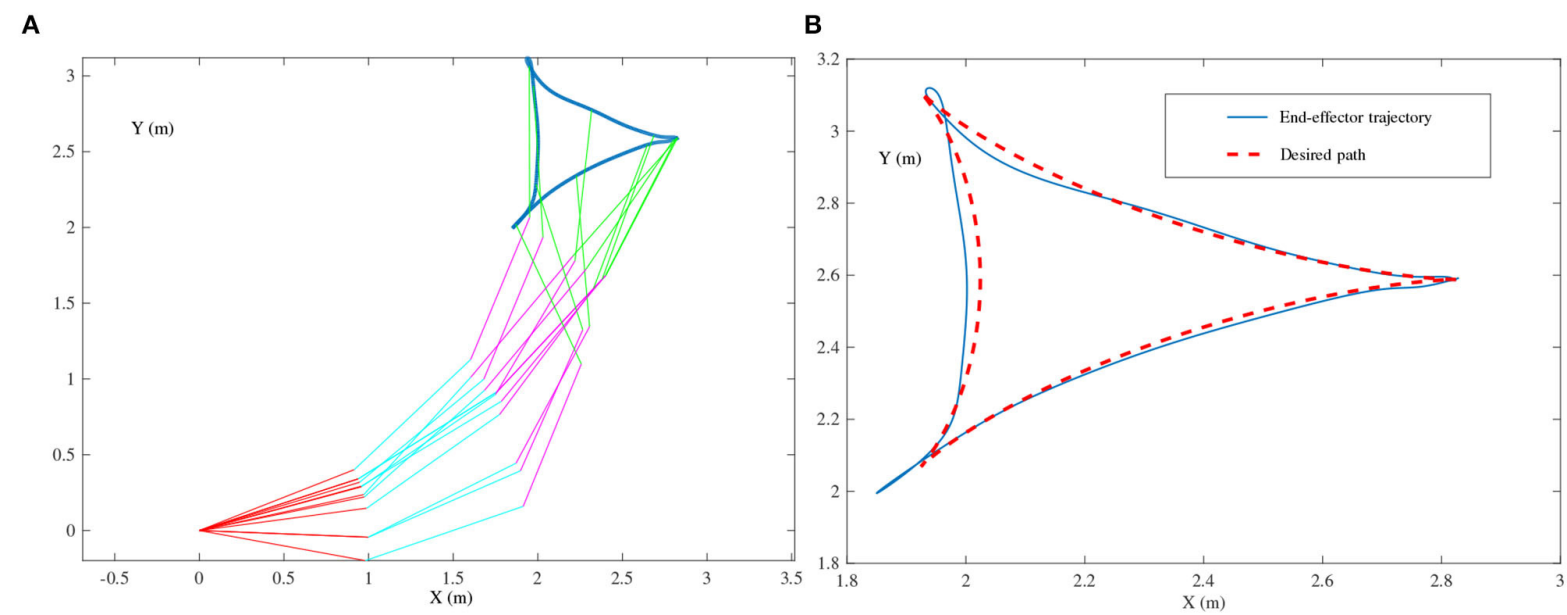
End-effector of the four-link planar robot manipulator tracking the tricuspid path synthesized by the noise-polluted kinematic control method in (29) using τ = 10, where single-harmonic noise is considered. **(A)** Simulated motion trajectories. **(B)** Path and trajectory.

**Figure 9:**
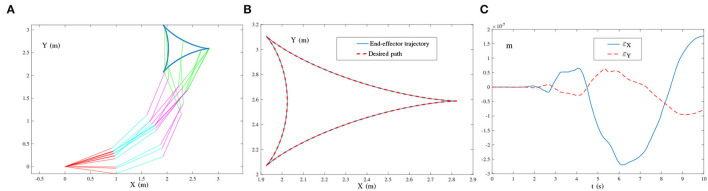
End-effector of the four-link planar robot manipulator tracking the tricuspid path synthesized by the ZNN-combined kinematic control method in (27) using τ = 10 and λ = 1, where single-harmonic noise is considered. **(A)** Simulated motion trajectories. **(B)** Path and trajectory. **(C)** End-effector positioning errors.

**Figure 10:**
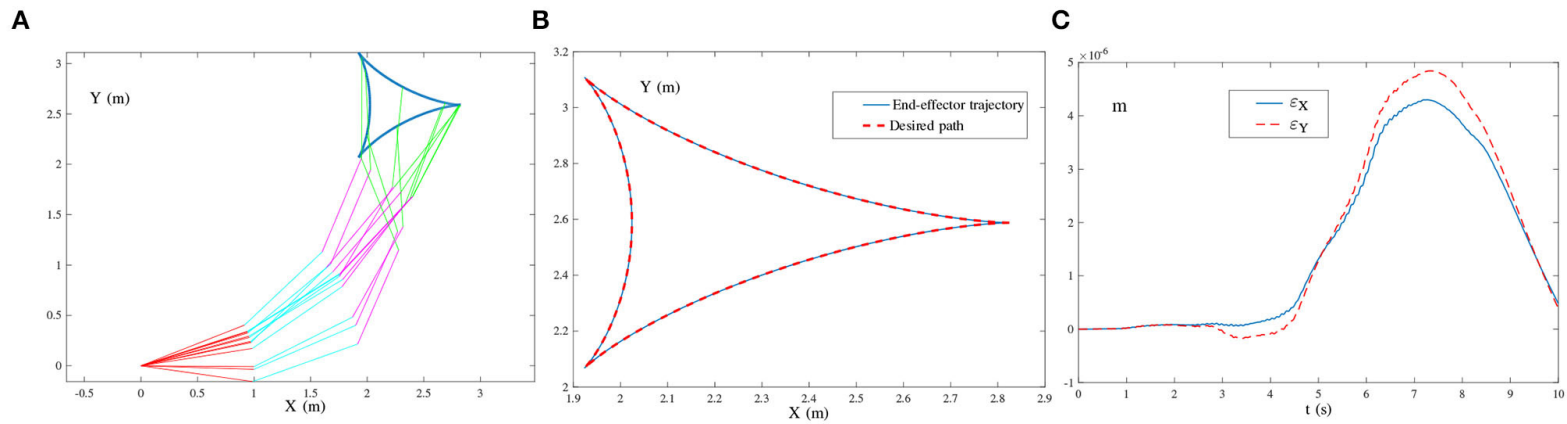
End-effector of the four-link planar robot manipulator tracking the tricuspid path synthesized by the ZNN-combined kinematic control method in (28) using τ = 10 and λ = 1, where multiple-harmonic noise is considered. **(A)** Simulated motion trajectories. **(B)** Path and trajectory. **(C)** End-effector positioning errors.

**Figure 11:**
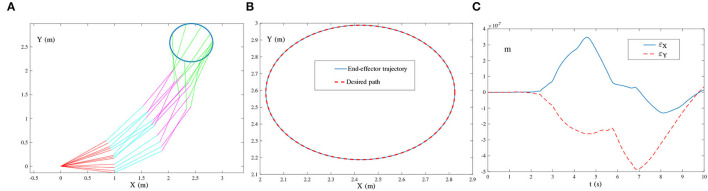
End-effector of the four-link planar robot manipulator tracking the circular path synthesized by the ZNN-combined kinematic control method in (28) using τ = 10 and λ = 1, where single-harmonic noise is considered. **(A)** Simulated motion trajectories. **(B)** Path and trajectory. **(C)** End-effector positioning errors.

**Figure 12:**
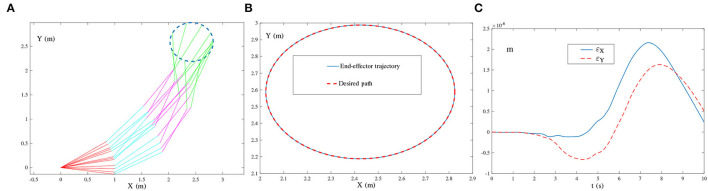
End-effector of the four-link planar robot manipulator tracking the circular path synthesized by the ZNN-combined kinematic control method in (28) using τ = 10 and λ = 1, where multiple-harmonic noise is considered. **(A)** Simulated motion trajectories. **(B)** Path and trajectory. **(C)** End-effector positioning errors.

1. Tricuspid path-tracking example: In this example, the presented ZNN-combined kinematic control methods in (27) and (28) are simulated for the four-link planar robot manipulator with its end-effector tracking a tricuspid path.
  ★ Single-harmonic noise: The ZNN-combined kinematic control method in (27) is investigated for the single-harmonic noise of $N\left(t\right)=\left[n_{ij}\left(t\right)\right]=\left[10\sin\left(2\pi t+1\right)\right]\in {\mathbb{R}}^{2\times 3}$, in which the harmonic frequency is *f* = 2π/(2π) = 1 Hz. For comparison, the following noise-polluted kinematic control method based on the original ZNN design in (2) is also simulated:
  (29) $$\begin{array}{l} \{\begin{array}{c} \ddot{\theta }\left(t\right)=B^{†}\left(t\right){\ddot{r}}_{\text{a}}\left(t\right),\\ B^{\text{T}}\left(t\right)B\left(t\right)Ẏ\left(t\right)={Ḃ}^{\text{T}}\left(t\right)-({Ḃ}^{\text{T}}\left(t\right)B\left(t\right)+B^{\text{T}}\left(t\right)Ḃ\left(t\right))Y\left(t\right)\\ -\tau \text{Ψ}(B^{\text{T}}\left(t\right)B\left(t\right)Y\left(t\right)-B^{\text{T}}\left(t\right))+O\left(t\right). \end{array} \end{array}$$
  Figure 8 presents the simulation results synthesized by (29) using τ = 10. The simulated end-effector trajectory of the robot manipulator does not match the desired tricuspid path, which means that (29) has failed to achieve the kinematic control of the robot manipulator due to the existence of harmonic noise. As shown in Figures 9A,B, the simulated end-effector trajectory is very close to the desired tricuspid path. Figure 9C shows that the maximal end-effector positioning error is <2 × 10^−7^ m, suggesting that the kinematic control is achieved successfully *via* (27) even if harmonic noise exists. These comparative results substantiate the effectiveness and noise-suppression capability of the ZNN-combined kinematic control method in (27) for the four-link planar robot manipulator.
  ★ Multiple-harmonic noise: The ZNN-combined kinematic control method in (28) is investigated for the multiple-harmonic noise:
  $$\begin{array}{l} Õ\left(t\right)=\left[{õ}_{ij}\left(t\right)\right]\\ =\left[10\sin\left(2\pi t+1\right)+10^{2}\sin\left(10\pi t+2\right)+10^{3}\sin\left(20\pi t+3\right)\right], \end{array}$$
  in which the harmonic frequencies are denoted by *f*_1_ = 1, *f*_2_ = 5, and *f*_3_ = 10 Hz. The corresponding simulation results synthesized by (28) are presented in Figure 10. The simulated end-effector trajectory is close to the desired tricuspid path, with the maximal positioning error being <5 × 10^−6^ m. Owing to the robustness of (28), this method is tolerant of multiple-harmonic noise, and the robot manipulator can track the desired tricuspid path successfully. The effectiveness of the ZNN-combined kinematic control method in (28) for the four-link planar robot manipulator is, thus, demonstrated.
2. Circular path-tracking example: In this example, the presented ZNN-combined kinematic control methods in (27) and (28) are simulated for the four-link planar robot manipulator with its end-effector tracking a circular path. Similarly, the following two harmonic noises are considered in the investigation of (27) and (28):
  $$\begin{array}{l} \{\begin{array}{c} O\left(t\right)=\left[o_{ij}\left(t\right)\right]=\left[10\sin\left(136\pi t+1\right)\right],\\ Õ\left(t\right)=\left[{õ}_{ij}\left(t\right)\right]=[10\sin\left(130\pi t+1\right)+10^{2}\sin\left(136\pi t+2\right)\\ +10^{3}\sin\left(140\pi t+3\right)]\in {\mathbb{R}}^{2\times 3}. \end{array} \end{array}$$
  The frequency of the single-harmonic noise approximates the bandwidth of the robot manipulator. The corresponding simulation results are illustrated in Figures 11, 12.

As shown in Figure 11, the simulated end-effector trajectory of the robot manipulator matches the desired circular path, and the maximal positioning error is less than 4 × 10^−7^ m. These results substantiate the successful application of the ZNN-combined kinematic control method in (27) to the robot manipulator. The results presented in Figure 11 also show that (27) has achieved robustness against single-harmonic noise, even if the noise frequency is nearly the same as the robot's bandwidth. Furthermore, the results presented in Figure 12 suggest that the path-tracking task has been successfully completed even with the presence of multiple-harmonic noise. The effectiveness of the ZNN-combined kinematic control method in (28) for the robot manipulator is further demonstrated.

In summary, the simulation results (i.e., Figures 8–12) prove that the presented ZNN-combined kinematic control methods in (27) and (28) are more effective for the robot manipulator under the presence of harmonic noise compared with the noise-polluted the kinematic control method in (29). More importantly, the results indicate the application prospect of the proposed HNTZNN models in (11) and (14) *via* dynamic matrix pseudoinverse.

## 7. Conclusion

In this article, by combining the harmonic signal dynamics and the Li activation function, two harmonic noises tolerant ZNN models with fast convergence have been proposed. The theoretical analysis demonstrates that the proposed HNTZNN models possess satisfactory convergence and robustness properties. Furthermore, an example has been given to verify its effectiveness in solving the dynamic matrix pseudoinverse under different harmonic noises. Besides, such two HNTZNN models have been applied to the kinematic control of the four-link planar robot manipulator. In summary, the corresponding experimental results substantiate the effectiveness, superiority, and application prospect of the proposed HNTZNN models even with the existence of harmonic noises. Future study may lie in studying the proposed ZNN models activated by different activation functions. Another future research direction is to extend the proposed ZNN models to other application fields.

## Data Availability Statement

The original contributions presented in the study are included in the article/supplementary material, further inquiries can be directed to the corresponding author/s.

## Author Contributions

BL and DG presented the scheme. BL and YW designed experiments and wrote the manuscript. YW and JL carried out experiments. JL and YH analyzed the experimental results. All authors contributed to the article and approved the submitted version.

## Funding

This study was supported in part by the National Natural Science Foundation of China (62066015 and 61962023), the Hunan Natural Science Foundation of China (2020JJ4511), the Research Foundation of Education Bureau of Hunan Province, China (20A396), and the Scientific Research Foundation of Jishou University (Jdy20063).

## Conflict of Interest

The authors declare that the research was conducted in the absence of any commercial or financial relationships that could be construed as a potential conflict of interest.

## Publisher's Note

All claims expressed in this article are solely those of the authors and do not necessarily represent those of their affiliated organizations, or those of the publisher, the editors and the reviewers. Any product that may be evaluated in this article, or claim that may be made by its manufacturer, is not guaranteed or endorsed by the publisher.
